# TOR Regulates Cell Death Induced by Telomere Dysfunction in Budding Yeast

**DOI:** 10.1371/journal.pone.0003520

**Published:** 2008-10-24

**Authors:** Haiyan Qi, Yongjie Chen, Xuan Fu, Chao-Po Lin, X. F. Steven Zheng, Leroy F. Liu

**Affiliations:** Department of Pharmacology, UMDNJ-Robert Wood Johnson Medical School, Piscataway, New Jersey, United States of America; Ordway Research Institute, United States of America

## Abstract

Telomere dysfunction is known to induce growth arrest (senescence) and cell death. However, the regulation of the senescence-death process is poorly understood. Here using a yeast dysfunctional telomere model *cdc13-1*, which carries a temperature sensitive-mutant telomere binding protein Cdc13p, we demonstrate that inhibition of TOR (Target of Rapamycin), a central regulator of nutrient pathways for cell growth, prevents cell death, but not growth arrest, induced by inactivation of Cdc13-1p. This function of TOR is novel and separable from its G1 inhibition function, and not associated with alterations in the telomere length, the amount of G-tails, and the telomere position effect (TPE) in *cdc13-1* cells. Furthermore, antioxidants were also shown to prevent cell death initiated by inactivation of *cdc13-1*. Moreover, inhibition of TOR was also shown to prevent cell death induced by inactivation of telomerase in an *est1* mutant. Interestingly, rapamycin did not prevent cell death induced by DNA damaging agents such as etoposide and UV. In the aggregate, our results suggest that the TOR signaling pathway is specifically involved in the regulation of cell death initiated by telomere dysfunction.

## Introduction

Telomere function has been linked to aging. In humans, telomere length has been correlated with lifespan [1]. In mice lacking telomerase activity, progressively shortened telomeres is associated with premature aging [2], while restoration of telomerase activity rescues this phenotype [3]. Due to limited telomerase activity, majority of human somatic cells exhibit progressive shortening of telomeres with each cell division. Eventually, the critically shortened telomeres lead to exposure of the natural double-strand breaks at the ends of chromosomes, followed by activation of DNA damage responses and induction of ATM-dependent growth arrest, a process termed replicative senescence. Following prolonged growth arrest, massive cell death occurs eventually. Cells undergoing senescence-death are characterized by polyploidy (>4N DNA in mammalian cells) and enlarged sizes [4]–[6]. It appears that the function of telomeres is to protect the chromosome ends from being recognized as damaged DNA.

In human, telomeres consist of repeats of TTAGGG/CCCTAA about 10 kb in length and are progressively shortened during aging [7], [8]. The very ends of telomeres are 3′ protruding single-stranded TTAGGG repeats about 150 bp in length, referred to as telomeric G-tails or 3′ G-rich overhangs [9]–[11]. The protection of telomeres is achieved by telomere binding proteins. For instance, TRF1 binds to the double-stranded TTAGGG/CCCTAA repeats, TRF2 binds to the junction of the double-stranded and single-stranded telomeric region, and POT1 binds to single-stranded telomeric repeats (G-tails). Expression of dominant-negative TRF2 results in activation of ATM-signaling pathway leading to growth arrest and apoptosis [12], while knockdown of POT1 leads to ATR-mediated DNA damage signaling [13], [14].

In budding yeast, *Saccharomyces cereviciea*, telomeres consist of TG_1-3_/C_1-3_A repeats about 300 bp in length. Single-stranded telomeric G-tails can be detected during the S-phase of the cell cycle and are bound by Cdc13p [15]–[19]. Deletion of telomerase or mutations in telomere binding protein Cdc13p leads to dysfunctional telomeres. Inactivation of Cdc13p at the non-permissive temperature 37°C in *cdc13-1* ts mutant cells is known to lead to deprotected telomeres, resulting in Rad9-mediated G2/M arrest followed by cell death [15]–[17].

Our previous studies have shown that inactivation of Cdc13p results in apoptosis-like cell death that is characterized by caspase activation, phosphatidylserine (PS) flipping and ROS production [20]. These apoptotic signals are suppressed in a mitochondrial ρ° mutant [20]. Furthermore, these apoptotic signals were also shown to depend on *MEC1,* the ATR/ATM homologue in budding yeast [20].

TOR (target of rapamycin), a member of the phosphatidylinositide-3 kinase-related kinase (PIKK) superfamily, integrates nutrient and energy signals in eukaryotes to regulate cell growth and cell sizes (see review by Wullschleger) [21]. Upon activation, TOR stimulates ribosomal biogenesis and global translation to promote cell growth through a number of downstream effectors. When inhibited by starvation or its specific inhibitor rapamycin, autophagy and antistress genes are activated [21]. The TOR signaling pathway has recently been shown to play an important role in the regulation of the aging process in several model systems [22]–[25]. Here we demonstrate a novel link between TOR and telomeres: inhibition of TOR prevents cell death induced by inactivation of Cdc13p.

### Inhibition of TOR suppresses cell death induced by inactivation of the yeast telomere binding protein Cdc13p

**Figure 1:**
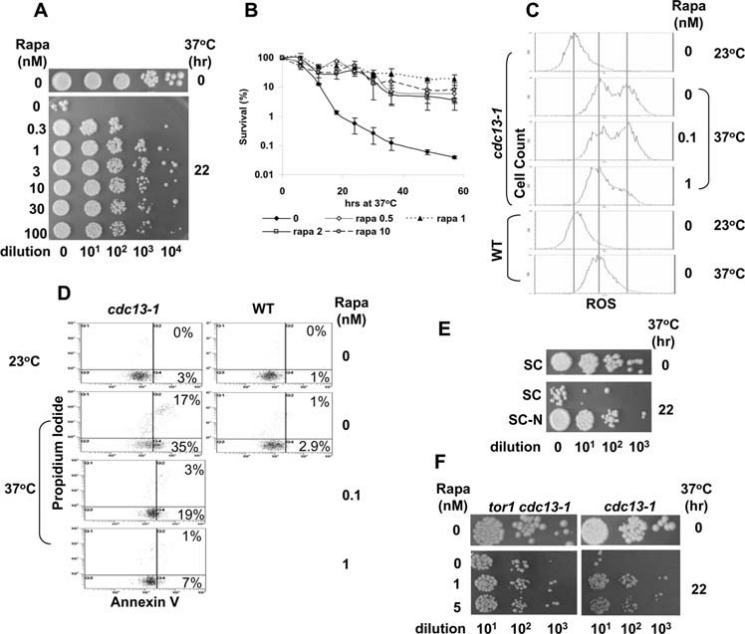
Rapa inhibits cell death induced by Cdc13p inactivation. (A) Colony formation assay. Fresh overnight culture of *cdc13-1* cells grown at 23°C was diluted into fresh YEPD in the presence of rapa (0 to 100 nM). Cells were incubated at 37°C for 0 and 22 hrs, and then 10-fold serially diluted and spotted on YEPD plates, followed by incubation at 23°C for 4 days. (B) Time-dependent decrease in cell survival. Cells were treated as described in (A). For every 6 hrs, an aliquot of cells were taken out and survived cells were measured using colony formation assay. The results were averages of triplicate experiments. (C) Dihydrorhodamine 123 staining for ROS. Treated *cdc13-1* cells (as in (A)) were stained with dihydrorhodamine 123, followed by FCAS analysis. (D) Annexin V staining for PS flipping. *cdc13-1* cells were treated as described in (A). Cells were then stained with PI and FITC-annexin V as described in Methods followed by FACS analysis. (E) Nitrogen starvation inhibits cell death induced by Cdc13p inactivation. The overnight culture in YEPD was diluted into fresh SC or SC-N medium. Colony formation assay was performed as in (A). (F) Colony formation assay using *tor1::kan cdc13-1*. Colony formation assay was performed as described in (A) except that the strain *tor1::kan cdc13-1* was used instead.

**Figure 2:**
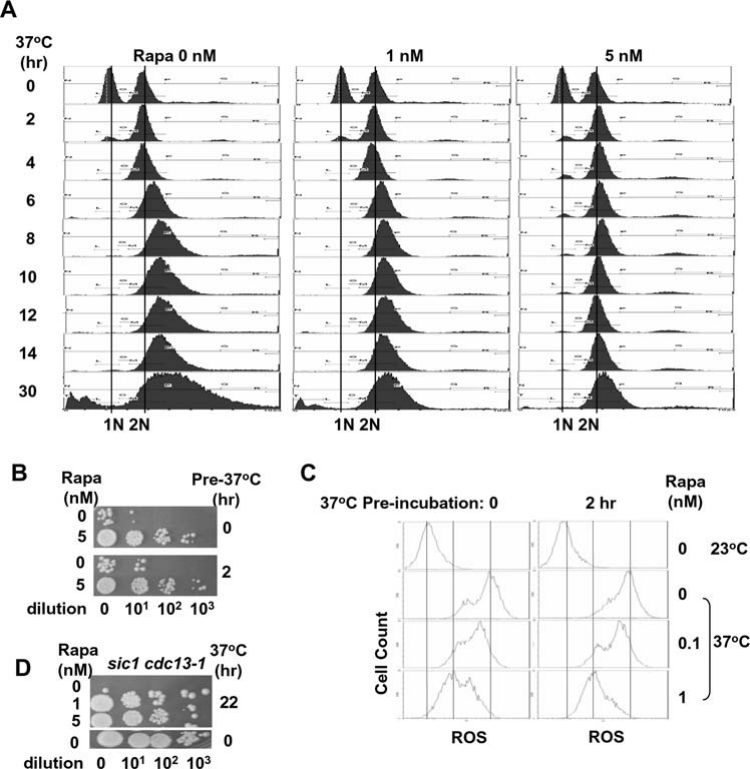
The cell death protective effect of rapa is independent of its G1 cell cycle effect. (A) *cdc13-1* cells in YEPD were treated with rapa (0, 1 and 5 nM) at 37°C for indicated time (0 to 30 hrs). Cells were then fixed and stained with PI (for DNA content), followed by FACS analysis. (B) *cdc13-1* cells in YEPD were cultured at the non-permissive temperature (37°C) for 0 and 2 hrs, followed by treatment with rapa (0, 0.1, 1 and 10 nM) at the same temperature for 22 hrs. Colony formation assay was then performed. (C) Cells obtained from (B) were also stained with dihydrorhodamine 123 for ROS measurement, followed by FACS analysis. (D) *sic1cdc13-1* cells were incubated at 37°C for 22 hrs followed by colony formation assay.

To test whether there is a connection between TOR and telomere dysfunction-induced cell death, a budding yeast temperature sensitive (ts) mutant strain, *cdc13-1,* was employed. Consistent with previous studies [20], inactivation of Cdc13p induced growth arrest followed by massive cell death one day later (Fig. 1 and 2). As shown in Fig. 1A (compare the first and the second rows) and 1B, incubation of *cdc13-1* cells (haploid) at the non-permissive temperature (37°C) for 22 hrs resulted in massive loss of viable cells as measured by the colony formation assay. Consistent with previous studies, cell death was manifested by appearance of apoptotic markers such as ROS production (Fig. 1C), increased PS flipping (Fig. 1D), and caspase activation (Fig. S1) [20]. Cell death was also accompanied by cell enlargement (Fig. S2) and hyperploidy (Fig. 2A). Interestingly, the TOR-specific inhibitor, rapamycin (rapa), effectively prevented the death of *cdc13-1* cells at the non-permissive temperature. Rapa at concentrations as low as 0.3 to 3.0 nM effectively inhibited cell death (Fig. 1A). As shown in Fig. 1B, after 60 hrs of incubation at the non-permissive temperature, cells treated with various concentrations of rapa survived at least 100-fold more than those without rapa treatment. It was noted that rapa also effectively inhibited apoptotic signals such as increased production of ROS (Fig. 1C), caspase activation (Fig. S1), and PS flipping (Fig. 1D). In addition, rapa also inhibited cell swelling (Fig. S2) and reduced the cell population with greater than 2N DNA content (Fig. 2A).

Rapa is known to bind at the interface between FKBP12 and TOR, resulting in TOR inhibition [26]. To rule out the possibility that the protective effect of rapa may come from inhibition of FKBP12 rather than TOR, a FKBP12 inhibitor, FK506, was used. FK506 is known to bind at the interface of FKBP12 and calcineurin [27]. As shown in Fig. S3A and S3B, FK506 neither protected *cdc13-1* cells in a colony formation assay, nor reduced ROS production as measured by dihydrorhodamine 123 staining.

To further confirm the involvement of TOR, the nitrogen starvation condition, known to inhibit TOR [28], was also employed. Nitrogen starvation by synthetic minimum medium (SC-N) lacking all nitrogen sources but containing the essential amino acids (leucine, tryptophan, uracil, and histidine for this yeast strain) prevented cell death induced by inactivation of Cdc13p (Fig. 1E). We also tested the involvement of TOR by employing a *tor1Δ cdc13-1* double mutant. As shown in Fig. 1F, there were more survivors in the *tor1Δ cdc13-1* double mutant than in the *cdc13-1* single mutant after 22-hr incubation at the non-permissive temperature. In this case, addition of rapa could not further improve survival of the double mutant (Fig. 1F). These results provide additional support for a role of TOR in regulating cell death induced by inactivation of the telomere binding protein Cdc13p.

### The role of TOR in telomere-initiated cell death is independent of its G1 function

Cdc13p has been shown to bind to telomeres primarily in S-phase [19]. TOR inhibition is known to prolong G1 and delay G1/S transition through its suppression on global translation [29]. The rapa's cell death-protective effect could be due to its effect on G1 inhibition which results in bypassing the requirement of Cdc13p in S-phase. However, as shown in Fig. 2A, rapa at 1 nM, which effectively prevented telomere-initiated cell death (see Fig. 1A), did not result in G1 accumulation, suggesting that the protective effect of rapa is unrelated to its G1 function.

To further rule out the possibility that the protective effect of rapa on cell death is linked to its G1 function, *cdc13-1* cells were pre-incubated at the non-permissive temperature (37°C) for two hrs to allow the majority of cells (greater than 95%) to exit G1 and accumulate at the G2/M phase (Fig. 2A). These G2/M-arrested cells were then treated with rapa for 22 hrs at 37°C. As shown in Fig. 2B and 2C, cell death and ROS production were prevented by rapa in these G2/M cells. Moreover, deletion of *SIC1* (an inhibitor of Cdc28-Clb kinase complexes that plays an important role in rapa-induced G1 block) [30], [31] did not affect the protective effect of rapa (Fig. 2D). Together, these results strongly suggest that the cell-death protective effect of rapa may represent a novel TOR function that is unrelated to its G1 function.

We have also observed that inactivation of Cdc13p led to an increase in DNA content. At 2–4 hrs post-inactivation, *cdc13-1* cells were initially arrested at the G2/M phase (2N DNA content). Prolonged inactivation (from 6 to 30 hrs) led to the accumulation of cells with DNA content progressively greater than 2N. Rapa treatment was shown to prevent the accumulation of cells with greater than 2N DNA content (Fig. 2A).

**Figure 3:**
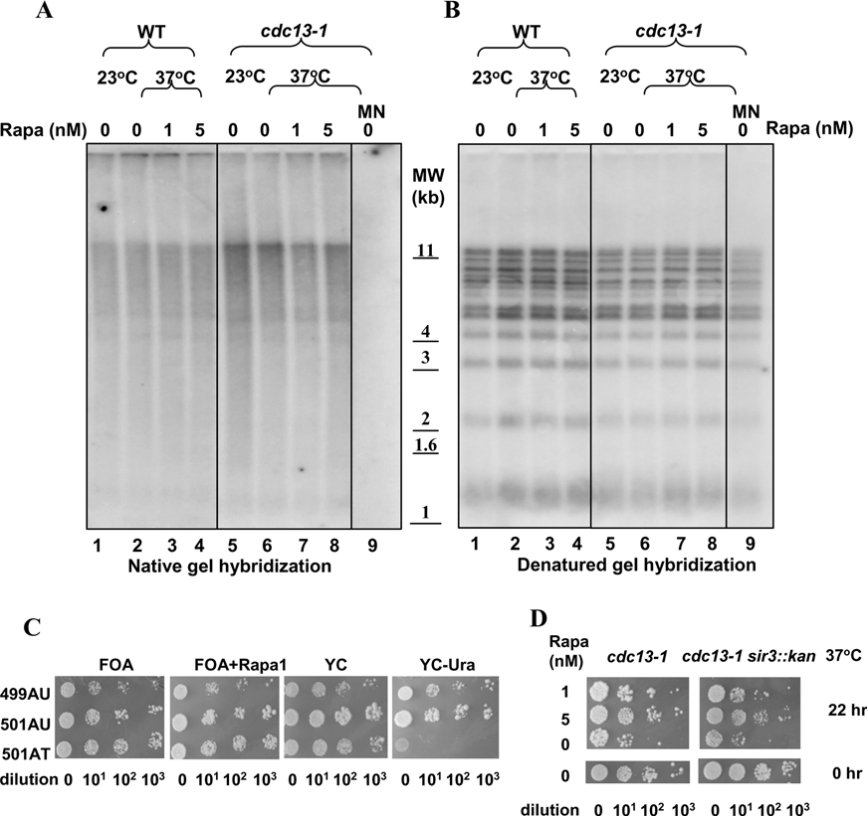
Rapamycin does not affect telomere state. (A). Rapamycin does not affect the amount of telomeric G-tails. Cells were incubated at the indicated temperatures for 22 hrs in the presence or absence of rapamycin. Genomic DNA was isolated, cut with XhoI and separated by 0.9% agarose gel. The gel was dried by a vacuum gel drier and probed by the ^32^P labeled telomeric C-strand probe. Lane 9 was the same as Lane 6 except treated with Moung bean nuclease (labeled as MN) for 30 min. (B). Rapamycin does not affect telomere length. The same gel in 3A was alkaline-denatured and probed with the same telomeric C-stand probe. (C). Rapamycin does not affect TPE. Both of 499AU (mat a) and 501AU (mat a/α) contains a *URA3* gene at the left telomere of chromosome 7, while 501AT has no functional *URA3* gene. Log phase cells were 10-fold diluted and spotted on plates as indicated. (D). *SIR3* deletion does not significantly affect the cell death, neither the rapamycin prevention effect. Cells were incubated at 37°C for indicated time, in the absence or presence of rapamycin. Cell surviving was monitored by colony formation assay.

It is possible that rapa may prevent telomere-initiated cell death through an effect on the protective state of the telomeres. However, rapa did not exert any significant effect on the entry to G2/M, suggesting that the state of unprotected telomeres upon Cdc13p inactivation is not affected. To provide further evidence, we monitored the telomeric G-tails, the telomere length and the telomere position effect (TPE) [32] in rapa-treated cells. As shown in Fig. 3A, the amount of telomeric G-tails, as monitored by non-denaturing in-gel hybridization using a ^32^P-labeled C-strand probe, remained the same before and after rapa treatment. After denaturation of the same gel, the telomere length was measured by Southern blotting using the same probe. As shown in Fig. 3B, rapa treatment did not change the telomere length. The TPE was also investigated using the yeast strains YPH499UT (haploid) and YPH501UT (diploid), both containing a *URA3* gene that is adjacent to the left telomere of chromosome 7 (7L-telomere) [33]. YPH501AT, an *ura3-52* mutant strain, served as a control. Silencing the *URA3* gene adjacent to the 7L-telomere (i.e. YPH499UT and YPH501 UT) or lack of Ura3p (i.e. YPH501AT) allows cells to form colonies on the plate containing FOA, which can be converted to a toxic product by functional Ura3p. As shown in Fig. 3C, rapa did not affect colony formation on FOA plates, suggesting that rapa does not influence TPE in these cells. Furthermore, deletion of Sir3p (Silent Information Regulator 3, a key player in maintaining a repressed chromatin structure near telomeres) [34] did not affect the protective effect of rapa on cell death induced by inactivation of cdc13-1p (Fig. 3D). These results suggest that rapa may not target the deprotected telomere state induced by inactivation of Cdc13p, but rather the downstream death pathway.

### Inhibition of TOR specifically prevents cell death triggered by dysfunctional telomeres

**Figure 4:**
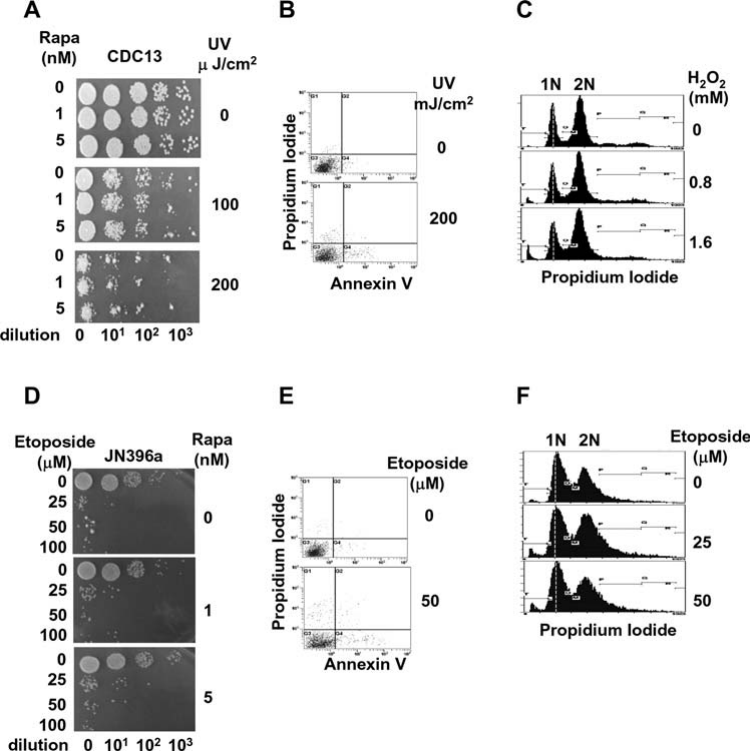
TOR dose not affect cell death induced by DNA damage agents significantly. (A–C) *CDC13* cells were diluted into fresh YEPD containing rapa (0, 1, and 10 nM), and then incubated at 23°C for 2 hrs. Cells were then 10-fold serially diluted and spotted onto YEPD plates. (A) Colony formation assay to measure surviving cells. Cells on plates were immediately irradiated with various doses of UV as indicated. Colonies were counted after five days of incubation in the dark at 23°C. (B) Annexin V binding assay for PS flipping. (C) DNA content analysis by FACS. (D–F) JN396a cells in YEPD were diluted into fresh YEPD containing rapa (0, 0.5 and 5 nM). Cells were then treated with etoposide (0, 25, 50 and 100 µM), followed by incubation at 23°C overnight. Cells were serially diluted and spotted onto YEPD plates for colony formation at 23°C (D), annexin V binding assay (E), and DNA content analysis by FACS (F).

Telomere deprotection is known to induce DNA damage signals [5], [12]. To determine whether rapa prevents cell death induced by DNA damaging agents, *cdc13-1* cells were treated with UV and etoposide (a DNA Top2 poison). As shown in Fig. 4, rapa (1 and 10 nM) did not affect cell death (assayed by colony formation) of yeast cells irradiated with UV (0, 100, and 200 µJ/cm^2^) (Fig. 4A), or treated with etoposide (0, 25, 50 and 100 µM) (Fig. 4D). It is interesting to note that different from inactivation of cdc13-1p, neither UV nor etoposide induced significant PS flipping (Fig. 4B, 4E,). In addition, neither UV nor etoposide increased the cell population with greater than 2N DNA content (Fig. 4C and 4G). These results suggest that rapa specifically prevents telomere-initiated cell death.

### Antioxidants prevent cell death induced by inactivation of Cdc13p

**Figure 5:**
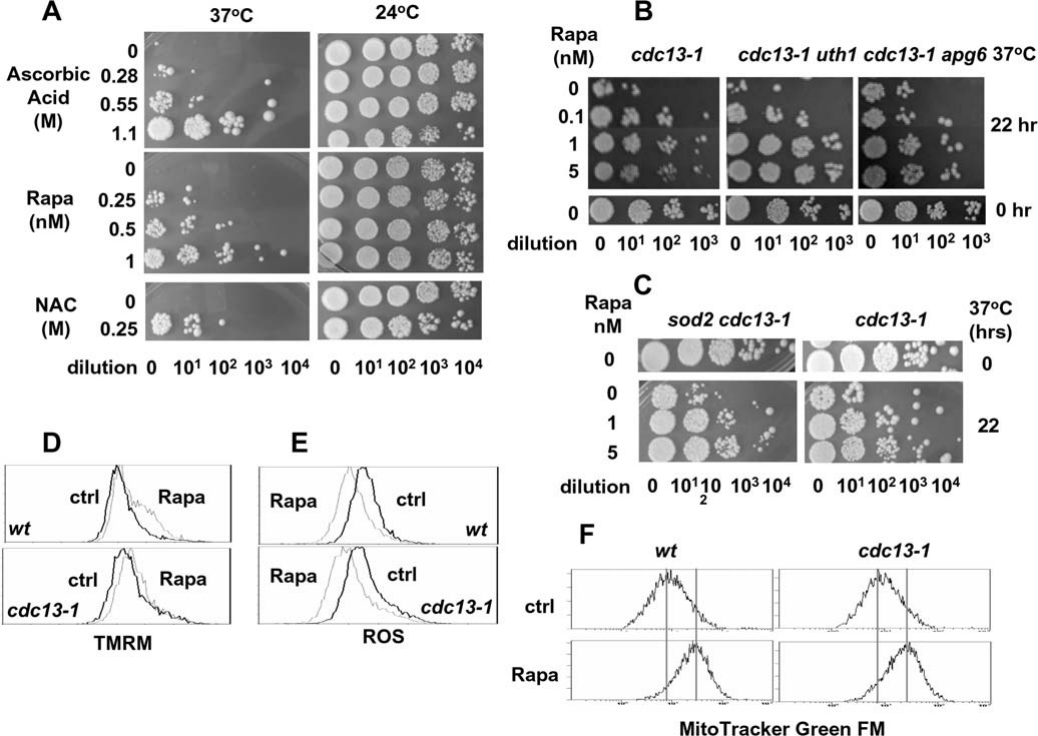
The role of ROS and mitochondria in cell death induced by inactivation of Cdc13p. (A) Antioxidants inhibit cell death induced by inactivation of Cdc13-1p. Cells were incubated at 37°C or 23°C for 22 hrs in the presence of indicated concentrations of vitamin C, NAC, and rapa. Cell viability was measured by colony formation assay. (B) The rapa protective effect on cell death initiated by dysfunctional telomeres in *cdc13-1* cells is not dependent on SOD2. *cdc13-1* and *sod2 cdc13-1* cells were incubated at 37°C for indicated time (hrs) with indicated concentrations of rapa. Colony formation assay was then performed as described in Methods. (C) The rapa protective effect on cell death initiated by dysfunctional telomeres in *cdc13-1* cells is not dependent on autophagy. Fresh overnight cultures of *cdc13-1*, *atg6 cdc13-1* and *uth1 cdc13-1* were diluted (1:11) into fresh YEPD medium in the presence and absence of rapa. Cells were then incubated at 37°C for 22 hrs, and cell viability was measured by colony formation assay. (D–F). Rapa increases mitochondrial membrane potential and reduces ROS production. YPH499 (wild type *CDC13*) and W13a (*cdc13-1*) cells were cultured in YEPD medium in the presence of rapa (0 and 2 nM) overnight at 30°C and 23°C, respectively. Mitochondrial membrane potential (D), ROS production (E) and mitochondrial mass (F) were measured as described in Methods.

As shown in Fig. 1C, inactivation of Cdc13p resulted in an elevated level of ROS that was abolished by rapa. To investigate the role of ROS in cell death upon inactivation of Cdc13p, the antioxidants, vitamin C and NAC (N-acetyl cysteine), were employed. As shown in Fig. 5A, both vitamin C and NAC prevented cell death initiated by inactivation of Cdc13p. The similar effect of rapa and antioxidants on preventing cell death suggests the possibilty that rapa may prevent telomere-initiated cell death through an antioxidant action.

It is known that inhibition of TOR stimulates MSN2/4, which in turn up-regulates Sod2p, the mitochondrial superoxide dismutase that reduces superoxide and protects cells against oxygen toxicity. However, deletion of SOD2 didn't affect the rapa protective effect (Fig. 5B), suggesting that the protective effect of rapa is not dependent on SOD2. Autophagy has been shown to be involved in the degradation of damaged mitochondria and could potentially affect cellular ROS [35]. To test the role of autophagy in the rapa protective effect, we deleted the *UTH1* gene [36] and the *ATG6* gene [37] in *cdc13-1* cells. As shown in Fig. 5C, deletion of *UTH1* or *ATG6* had no significant effect on cell death, ROS production, or the rapa protective effect in *cdc13-1* cells (at the non-permissive temperature), suggesting that cell death triggered by Cdc13p inactivation and the rapa-protective effect are not dependent on autophagy.

### Rapa increases mitochondrial membrane potential and reduces ROS production

ROS is primarily generated in mitochondria during oxidative phosphorylation for ATP production. Our previous studies have shown that mitochondria play an important role in apoptotic death induced by inactivation of Cdc13p [38].We therefore tested whether rapa affects mitochondrial function. As shown in Fig. 5D, rapa increased overall mitochondrial membrane potential measured by TMRM, a mitochondrial dye sensitive to mitochondrial membrane potential, in wt cells cultured at 30°C and *cdc13-1* cells cultured at the permissive temperature, 23°C. Under the same growth conditions, rapa also reduced ROS production measured by dihydrorhodamine 123 (Fig. 5E). In addition, rapa increased mitochondrial mass as monitored by FACS analysis of MitoTracker Green FM-stained cells (Fig. 5F). These results suggest that rapa may prevent cell death through regulating mitochondrial functions.

### Rapamycin suppresses cell death induced by inactivation of the essential subunit of telomerase Est1p

**Figure 6:**
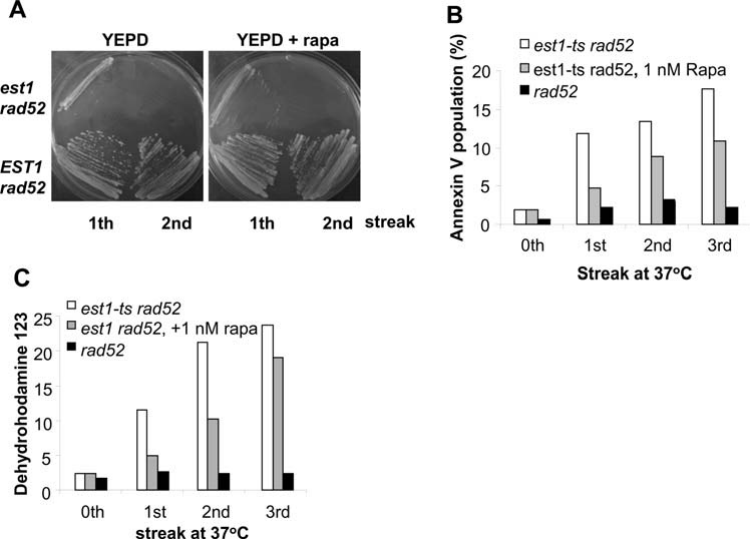
Rapamycin suppresses cell death induced by telomerase inactivation. (A) Rapamycin does not affect senescence. Fresh overnight cultures of *rad52* and *rad52 est1-ts* at 23°C (0^th^ streak) were streaked to YEPD plates containing 0 or 1 nM rapa (1^st^ streak). Cells were then incubated at 37°C for 2 days. Patches of cells were transferred (re-streaked) to fresh plates and incubated at 37°C for 2 days (2^st^ streak). (B–C) Rapamycin suppresses apoptotic death induced by inactivation of Est1p. Cells were treated as in (A). Large amount cells from 2^nd^ streak were transferred to fresh plates and incubated for 2 days at 37°C (3^rd^ streak). Patches of cells from each “streak” were collected and used for annexin V binding (B) and ROS production (C). PI-negative-FITC-positive cells represent apoptotic population and were used for plotting in (B).

To test whether the protective effect of rapa on cell death is telomere-specific, a budding yeast temperature-sensitive (ts) mutant strain, *est1-ts,* defective in the essential telomerase subunit Est1p [39], was employed. This strain exhibits progressive shortening of telomeres at 37°C, but not at 30°C or lower. Similar to inactivation of Cdc13p, inactivation of Est1p also results in G2/M arrest [6]. To minimize the complication caused by alternative telomere lengthening through Rad52-dependent recombination [40], a *rad52 est1-ts* double mutant (haploid strain) was used. Fresh overnight cultures of *est1-ts* and *est1-ts rad52* cells (cultured at 23°C) were streaked on YPED or YEPD+1 nM rapa plates and incubated at 23°C and 37°C (the non-permissive temperature). Patches of cells were then transferred (by streaking) to fresh plates every two days and incubated at 23°C and 37°C. *est1-ts rad52* cells consistently exhibited senescence (very slow growth on plates) without survivors by the 2^nd^ streak at 37°C in the presence or absence of rapa (Fig. 6A). Patches of cells were collected from plates at each transfer (streak) and subjected to FACS analysis for PS flipping (annexin V staining) and ROS production (dehydrorhodamine123 staining). As shown in Fig. 6 (B and C), the apoptotic population of *est1* cells (indicated by cell populations with elevated PS flipping and ROS production) increased significantly with successive transfers (streaks) at 37°C. The presence of rapa (1 nM) was shown to significantly suppress PS flipping and ROS production (Fig. 6B and 6C). These results suggest that rapa also suppresses cell death initiated by dysfunctional telomeres due to inactivation of the essential telomerase subunit Est1p.

## Discussion

We have previously demonstrated that inactivation of Cdc13p induces cell death that is characterized by apoptotic markers such as caspase activation, PS flipping, and ROS production [20]. In the current studies, we show that inhibition of TOR prevents the cell death induced by inactivation of Cdc13p based on both colony formation assay and measurement of various apoptotic markers. The protective effect of rapa is specific for telomere-initiated cell death since cell death induced by DNA damaging agents such as UV and etoposide (a prototypic topoisomerase II poison) [41] is minimally affected by rapa, but cell death initiated through inactivation of the essential telomere subunit 1 (*EST1*) is also prevented by rapa.

We have shown that the TOR-mediated protection of telomere-initiated cell death is novel and is also separable from its G1 regulatory function. The concentrations of rapa (1 nM and 5 nM) used in the current study are significantly lower than those required for G1 inhibition (Fig. 2). In addition, *cdc13-1* cells pre-arrested at the G2/M phase were also protected from death by rapa, again ruling out the G1 growth inhibition as a mechanism for the rapa's protective effect. In support of this conclusion, deletion of *SIC1* [30], [31] did not affect the protective effect of rapa (Fig. 2D). It is possible that low doses of rapamycin affect some signaling pathways of the TOR1 unrelated to its G1 function.

Interestingly, inhibition of TOR does not appear to be involved in modulating DNA damage signals under our experimental conditions based on the following observations: (1) Rapa does not change the state of telomeres measured by telomere length, the amount of telomeric G-tails and telomere position effect. (2) Rapa has no significant effect on cell death induced by UV, or etoposide. (3) Upon inactivation of Cdc13p, the entry into G2/M growth arrest, which is DNA damage checkpoint-dependent (e.g., Mec1, Rad9), is not affected by rapa. It appears that TOR regulates the downstream cell death pathway, but not the initial DNA damage signals induced by dysfunction telomeres in *cdc13-1* cells.

Our results have shown that the antioxidants, vitamin C and NAC, can also prevent cell death in *cdc13-1* cells, suggesting that ROS may play an important role in telomere-initiated cell death. We have therefore considered the possibility that rapa may prevent telomere-initiated cell death through an anti-oxidative pathway. Rapa may possibly control oxidative stress through multiple mechanisms. First, inhibition of TOR can activate an anti-stress defense system (including Sod2p) through Mns2/4 [42]. Second, inhibition of TOR may stimulate autophagy to degrade damaged mitochondria [35] and thereby regulates oxidative stress. Third, inhibition of TOR may regulate mitochondrial function directly and thereby control ROS production, since TOR has been reported to localize in mitochondria [43]. Our results show that rapamycin can improve mitochondrial membrane potential, increase mitochondrial mass and reduce ROS production. However, the protective effect of rapa is independent of Sod2p and autophagy. It is likely that inhibition of TOR prevents cell death triggered by inactivation of Cdc13p through multiple pathways.

It has been shown that chronologically aged budding yeast cells exhibit apoptotic markers and increased sizes [44]. In addition, inhibition of TOR have been shown to extend chronological lifespan of budding yeast [45]. Interestingly, extension of the chronological lifespan in budding yeast also appears to go through the mitochondrial respiration pathway [46]. It is likely that the death pathway in chronological aged yeast cells is similar to that caused by dysfunctional telomeres induced by inactivation of Cdc13p.

## Methods

### Yeast strains

W13a (*MATa cdc13-1 his7 leu2-3, 112 ura3-52 trp1-*289), W13α and its isogenic wild type strain *CDC13* (*MATα CDC13 his7 leu2-3, 112 ura3-52 trp1-*289) were obtained from Dr. Virginia A. Zakian (Princeton University, Princeton, NJ). *tor1* (*MATa his3D1 Leu2D0 ura3D0 met15D0 tor1::Kan*), *sic1*, *sod2, sir3* and its isogenic wild type BY4741 (*MATa his3D1 leu2D0 met15D0 ura3D0*) were obtained from a deletion library from Invitrogen (Carlsbad, California). *cdc13-1 tor1* and *cdc13-1 sod2* double mutant strains were generated by mating the single deletion mutant from the deletion library with W13α, followed by sporulating the diploid and selecting for temperature-sensitive and G418 (200 µg/ml)-resistant colonies. *cdc13-1 sir3, cdc13-1 apg6* and *cdc13-1 uth1* were made by replacing *APG6* or *UTH1* with the *KanMX4* gene in W13a background using a PCR-based gene replacement method[47]. JN394 (*MATa ura3-52 leu2 trp1 his7 ade2 ise2 rad52:LEU2*), an etoposide-permeable strain, was obtained from Dr. John Nitiss (St. Jude Children's Research Hospital, Memphis, TN). YPH499 (*MATa ade2-101, trp1-63, his3-200, leu2-1 ura3-52 lys2-801*), YPH499AU (*MATa ade2-101, trp1-63, his3-200, leu2-1 ura3-52 lys2-801 URA3* at a 7L telomere), YPH501AU (*MATa/α ade2-101, trp1-63, his3-200, leu2-1 ura3-52 lys2-801 URA3* at a 7L telomere) and YPH501AT (*MATa/α ade2-101, trp1-63, his3-200, leu2-1 ura3-52 lys2-801 ADE2* at a 5R telomere) were generous gifts from Dr. Virgina A Zakian.

### Inactivation of Cdc13p

Fresh overnight cultures of *cdc13-1* in YEPD were diluted (1:11) into YEPD, SC (0.67% yeast nitrogen base without amino acids, 0.1% amino acid mixture, 2% glucose), or SC-N (0.67% yeast nitrogen base without amino acids and (NH_4_)_2_SO_4_, 2% glucose, plus a mixture of 100 mg/L each of histidine, leucine, tryptophan and uracil). Cells were then incubated at 37°C (non-permissive temperature for W13a) overnight (22 hrs in most cases) in the presence or absence of rapamycin.

### Colony formation assay

Cells were 10-fold serially diluted in H_2_O in 96-well plates and spotted (5 µl from each dilution: 10^0^, 10^1^, 10^2^, 10^3^, 10^4^, and 10^5^) on YEPD plates, which were then incubated at 23°C for 4 days for colony formation.

### DNA content analysis

Cells were first fixed with 50% ethanol at −20°C, and then digested with 0.2 mg/ml RNase A in 50 mM Tris pH 7.6 at 37°C overnight. They were next washed with 50 mM Tris pH 7.6 and treated with 40 µg/ml proteinase K at 55°C for 2 hrs. After washing with PBS, cells were stained with 100 µg/ml propidium iodide for 20 min in the dark prior to FACS analysis.

### Phosphatidylserine (PS) flipping

Cells were resuspended in PBS buffer containing 1.1 M sorbitol and 2 mg/ml Zymolyase, and incubated at 37°C for 20 min. Cells were then stained with annexin V-FITC and propidium iodide (PI) (BD Biosciences Pharmingen) in PBS containing 1.1 M sorbitol, followed by FACS analysis. 10,000 cells were analyzed for each sample. Under this condition, PI-negative population represents intact cells. PI-negative-FITC-positive cells represent apoptotic population, while PI-positive-FITC positive cells represent late apoptotic or necrosis population.

### ROS production

The ROS level was measured by dihydrorhodamine 123 (Molecular Probe) which was converted to fluorescent rhodamine 123 upon oxidation. Cells were subsequently incubated with 5 µg/ml dihydrorhodamine 123 in YEPD for 1 hrs prior to FACS analysis. 10,000 cells were analyzed for each sample.

### In-gel hybridization

Yeast genomic DNA was isolated using MasterPure DNA purification kit from Epicentre Biotechnologies (Madison, WI). About 5 µg DNA was digested with *XhoI* for 4 hrs and then separated on 0.9% agarose gel by electrophoresis with 0.5xTBE buffer (10xTBE: 0.91% tetra NaEDTA, 5.5% Boric acid, 10.3% Tris pH8.4). Gel was placed between two 3MM paper and then dries using a vacuum gel dryer at room temperature 25°C for 40 min, followed by socked in dH_2_O for 2 min to remove 3 MM paper.

For Non-denaturing hybridization, gel was incubated overnight with pre-hybridization buffer containing 6xSSC, 0.3% SDS 10% formamide, 5 mM Na_2_HPO_4_/NaH_2_PO_4_ pH6.5 at room temperature 25°C. To make the C1-3A probe that specifically hybridizes to the telomeric G-tails, plasmid pCT300 digested with *EcoR* I and *Kpn* I and the telomeric fragment was gel-purified. 25 ng of this fragment was α-^32^P dCTP labeled for 15-30 min. After denaturation by boiling at 100°C for 5 min, the C_1-3_A probe was added to pre-hybridization buffer to make the hybridization buffer. The gel was then incubated with the hybridization buffer overnight at 25°C, followed by extensive washes: with wash buffer I containing 2x SSC and 0.1% SDS for 1 hr, wash buffer II containing 0.1x SSC and 1% SDS for 1 hr and prehybridization buffer for1 hr. This wash cycle was repeated 1–2 times to remove unspecific binding completely. The gel was then exposed to X-ray film.

For denatured hybridization, the gel was denatured with denaturing buffer containing 0.5M NaOH and 1.5M NaCl for 20 min, and neutralized with neutralization buffer containing 0.5M Tris pH7.0 and 3 M NaCl for 20 min. The denatured gel was then hybridized with C1-3 probe described above.

### Telomere position effect

YPH499AU and YPH501AU (Gift from Dr. Virginia A. Zakain were employed, in which URA3 gene was placed near a telomere (Chromosome 7 left arm) [33]. YPH500AT, which don't have a functional *URA3* gene, was also used as a negative control. Expression of *URA3* is necessary to support growth on medium lacking uracil. However, cells producing Ura3p cannot grow in the presence of 5-FOA, because it is converted into a toxic substance by the *URA3* gene product. Colony formation on YC+5-FOA is reduced due to TPE compared to that on YC and YC-Ura plates.

### Measurement of mitochondrial membrane potential and mitochondrial mass

For measuring mitochondrial membrane potential, treated cells were stained 5 µM TMRM (tetramethylrhodamine, methyl ester, perchlorate, from Invitrogen) for 15 min at dark, followed by FACS analysis. For measuring mitochondrial mass, cells were fixed with 60% ethanol at 4°C overnight. After wash with PBS, cells were incubated with 0.1 ng/ml MitoTracker Green FM (Invitrogen) diluted in PBS at Dark for 15 min. Cells were then analyzed by FACS. 10,000 cells per sample were analyzed.

### Inactivation of telomerase and senescence assay

Log phase *est1-ts rad52::URA3* and *EST1 rad52::URA3* cells cultured at 23°C were diluted (1 to 10) into fresh YEPD medium (1% yeast extract, 2% Bacto-peptone and 2% glucose, supplemented with 100 mg/l tryptophan and uracil) containing 0 and 1 nM rapamycin and incubated at 37°C (to inactivate telomerase). After 24-hr growth, cells were diluted again (1 to 10) and cultured at 37°C. This dilution/growth cycle at 37°C was repeated daily for seven days. Cell samples were collected daily after 24 hr growth at 37°C for cell density measurement and OD_595_ values were plotted against total time incubated at 37°C. Aliquots of cells were 10-fold serially diluted and spotted on YEPD plates for colony formation at 23°C. Aliquots of cells were also analyzed for DNA content, PS flipping and ROS production as described below [20]. Although recombination was largely suppressed by deleting the *RAD52* gene, *est1-rad52::URA3* cells cultured in liquid medium still frequently yielded random survivors. We routinely cultured and analyzed ten isolates from each strain for senescence assay. Only those data generated from isolates that did not show random survivors after 7-day culturing at 37°C were used.

## Supporting Information

Figure S1 Rapa inhibits caspase activation in cdc13-1 cells. Log phase cdc13-1 cells (OD595 about 0.2) cultured at 23°C were treated with indicated concentrations of rapa and incubated at 23°C or 37°C overnight, or at 80°C for 5 min. Cells were stained with 12 mM of FITC-VAD-FMK and 1 mg/ml propidium iodide (PI) for 20 min, washed with PBS and subjected to FACS analysis. The bottom panel: cells (80°C, 5 min ) were stained with FITC and PI instead of FITC-VAD-FMK and PI.
(10.36 MB TIF)

Figure S2 The effect of rapamycin on cell sizes. Log phase cdc13-1 cells were incubated at 37°C for 0, 5 or 24 hrs in the presence and absence of 4 nM rapamycin. 10,000 cells from each sample were counted by FACS analysis. Averages of cell sizes are represented by the X-means of the forward scatter.
(7.80 MB TIF)

Figure S3 FK506 fails to protect telomere-initiated cell death. a. Colony formation assay. cdc13-1 cells were cultured in YEPD medium overnight at room temperature. The overnight culture was then diluted into fresh YEPD and incubated at 37°C overnight. Cells were then 10-fold serially diluted and spotted on YEPD plates for colony formation at 23°C. b. Dihydrorhodamine 123 staining for ROS. As in a, cdc13-1 cells in YEPD were cultured overnight at 37°C and then stained with dihydrorhodamine 123 and PI followed by FACS analysis.
(11.07 MB TIF)
