# Supplementary figures and images for: TOR Regulates Cell Death Induced by Telomere Dysfunction in Budding Yeast
Source: PLoS One. 2008 Oct 24;3(10):e3520. doi: 10.1371/journal.pone.0003520 (PMC2567032; doi:10.1371/journal.pone.0003520)

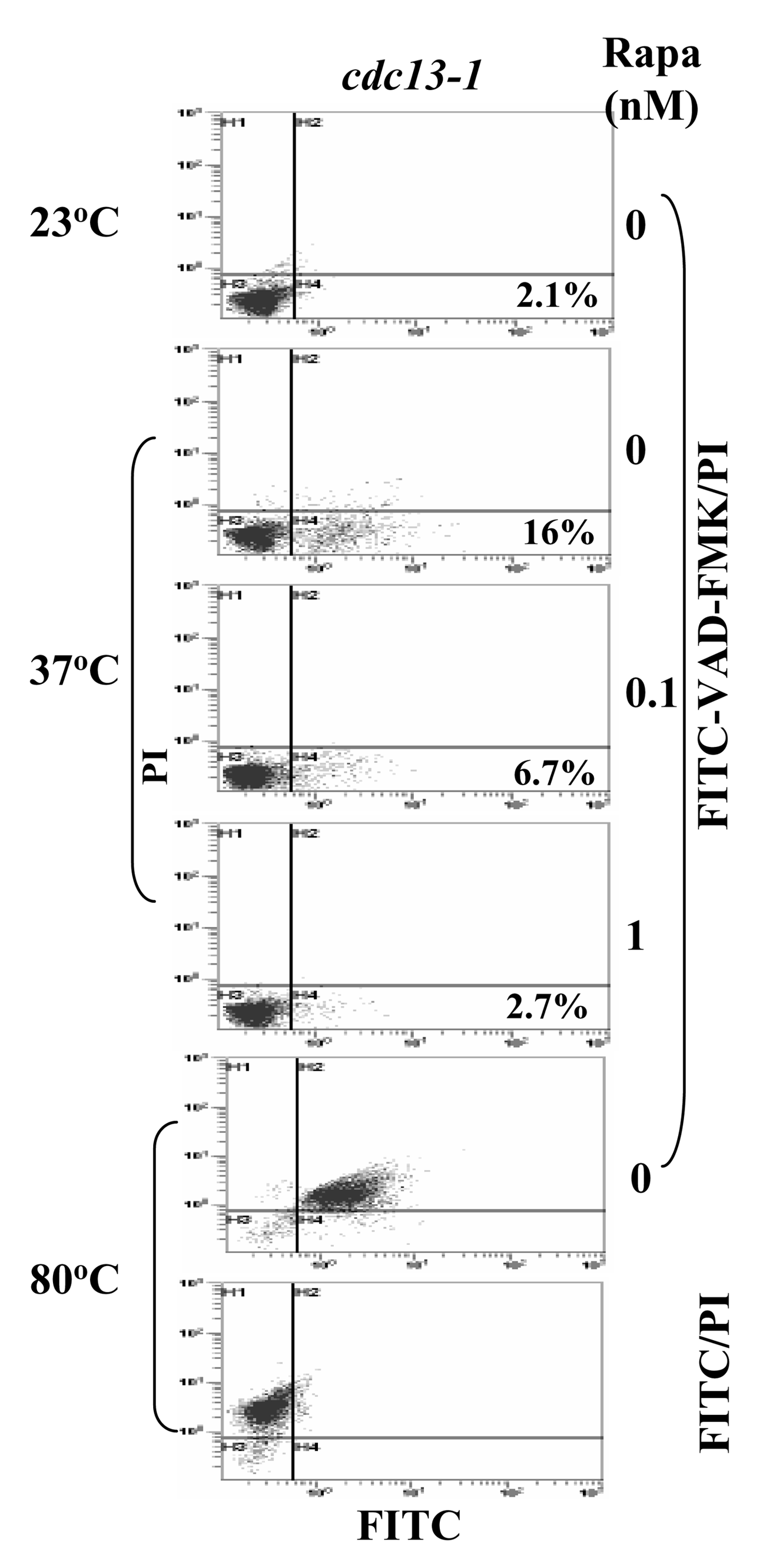

Supplement: Figure S1 — Rapa inhibits caspase activation in cdc13-1 cells. Log phase cdc13-1 cells (OD595 about 0.2) cultured at 23°C were treated with indicated concentrations of rapa and incubated at 23°C or 37°C overnight, or at 80°C for 5 min. Cells were stained with 12 mM of FITC-VAD-FMK and 1 mg/ml propidium iodide (PI) for 20 min, washed with PBS and subjected to FACS analysis. The bottom panel: cells (80°C, 5 min ) were stained with FITC and PI instead of FITC-VAD-FMK and PI. (10.36 MB TIF) [file pone.0003520.s001.tif]

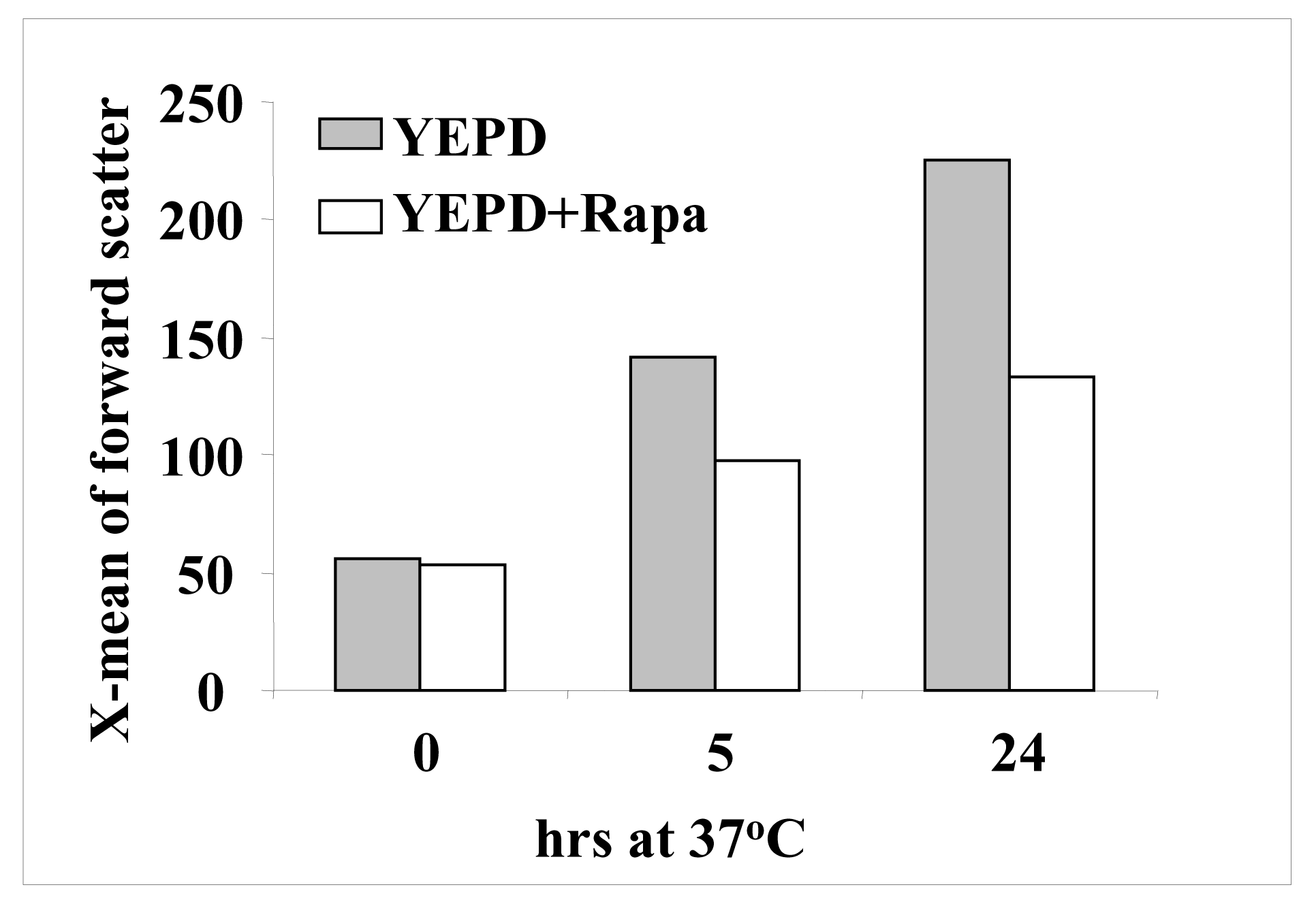

Supplement: Figure S2 — The effect of rapamycin on cell sizes. Log phase cdc13-1 cells were incubated at 37°C for 0, 5 or 24 hrs in the presence and absence of 4 nM rapamycin. 10,000 cells from each sample were counted by FACS analysis. Averages of cell sizes are represented by the X-means of the forward scatter. (7.80 MB TIF) [file pone.0003520.s002.tif]

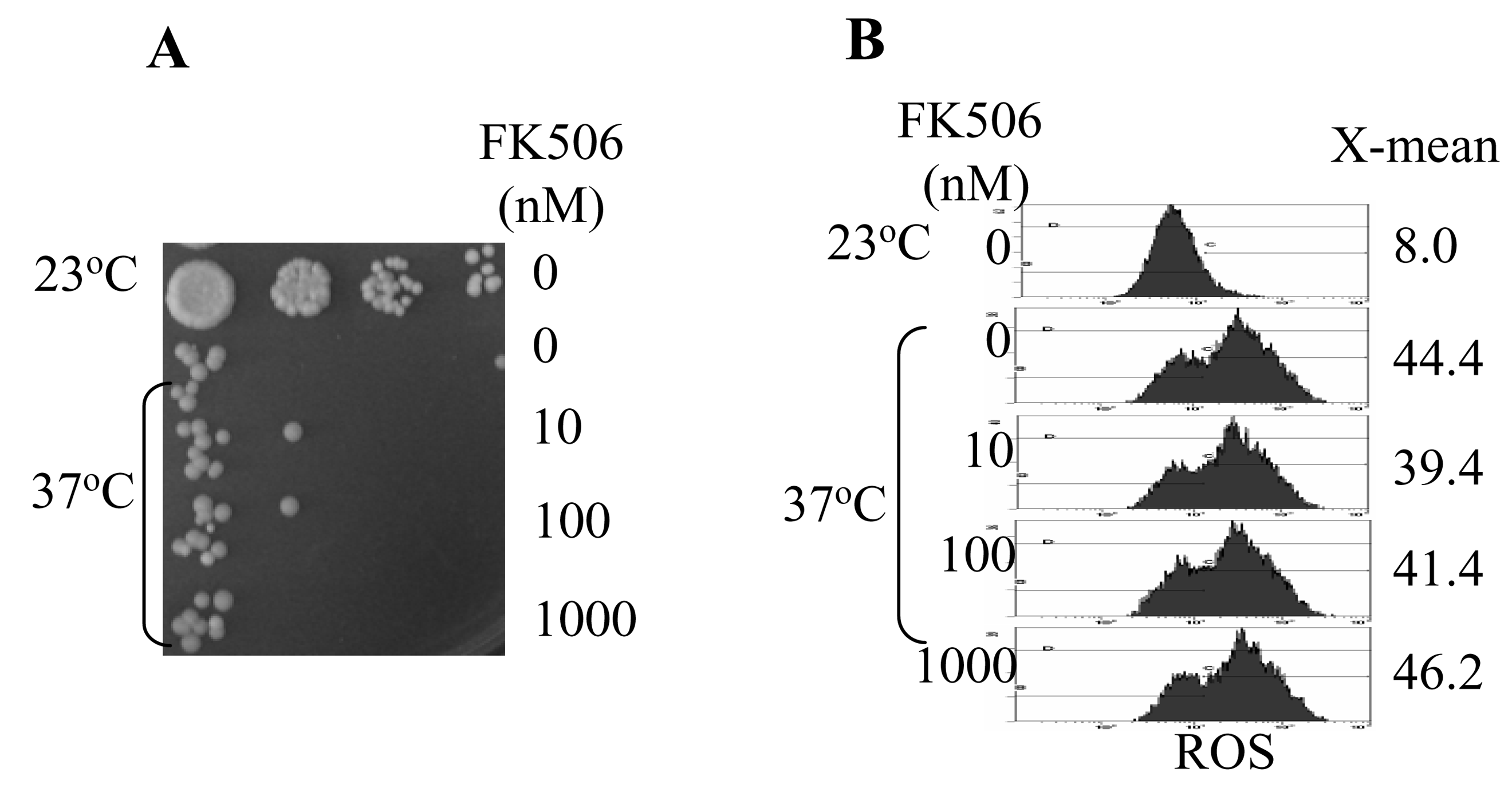

Supplement: Figure S3 — FK506 fails to protect telomere-initiated cell death. a. Colony formation assay. cdc13-1 cells were cultured in YEPD medium overnight at room temperature. The overnight culture was then diluted into fresh YEPD and incubated at 37°C overnight. Cells were then 10-fold serially diluted and spotted on YEPD plates for colony formation at 23°C. b. Dihydrorhodamine 123 staining for ROS. As in a, cdc13-1 cells in YEPD were cultured overnight at 37°C and then stained with dihydrorhodamine 123 and PI followed by FACS analysis. (11.07 MB TIF) [file pone.0003520.s003.tif]
